# Supplementary figures and images for: EBV infection outcomes determined by monocyte and TREG-driven immune dynamics in an ex vivo pbmc model
Source: PLoS Pathog. 2026 Mar 20;22(3):e1013746. doi: 10.1371/journal.ppat.1013746 (PMC13029685; doi:10.1371/journal.ppat.1013746)

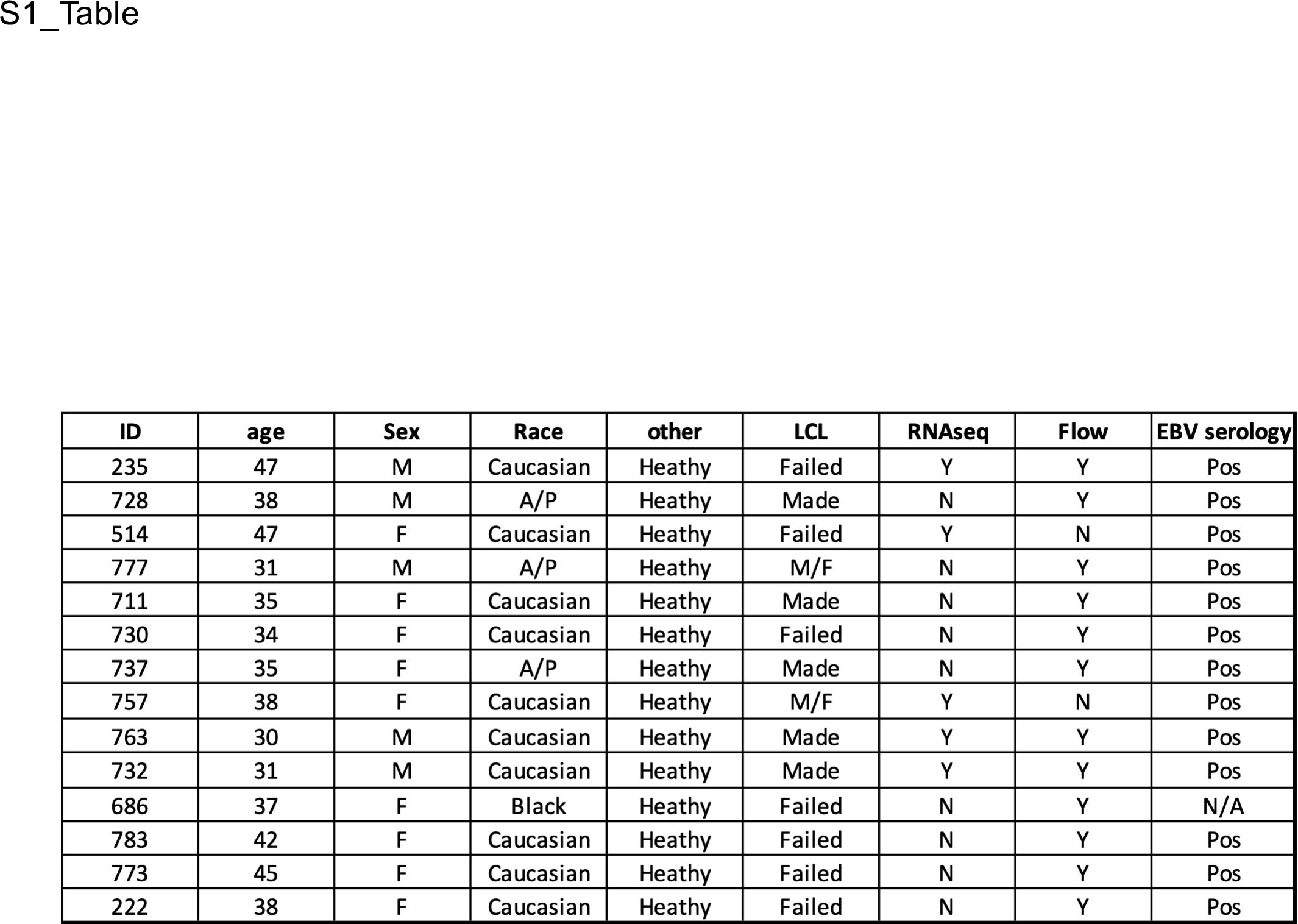

Supplement: S1 Table — Summary of donor characteristics used in EBV infection studies. The table includes donor ID, age, sex, race, and health status, along with experimental annotations indicating whether lymphoblastoid cell lines (LCLs) were successfully generated (Made, Failed, or M/F for mixed outcomes). Columns also denote availability of RNA-seq and flow cytometry data, as well as EBV serostatus. (TIF) [file ppat.1013746.s001.tif]

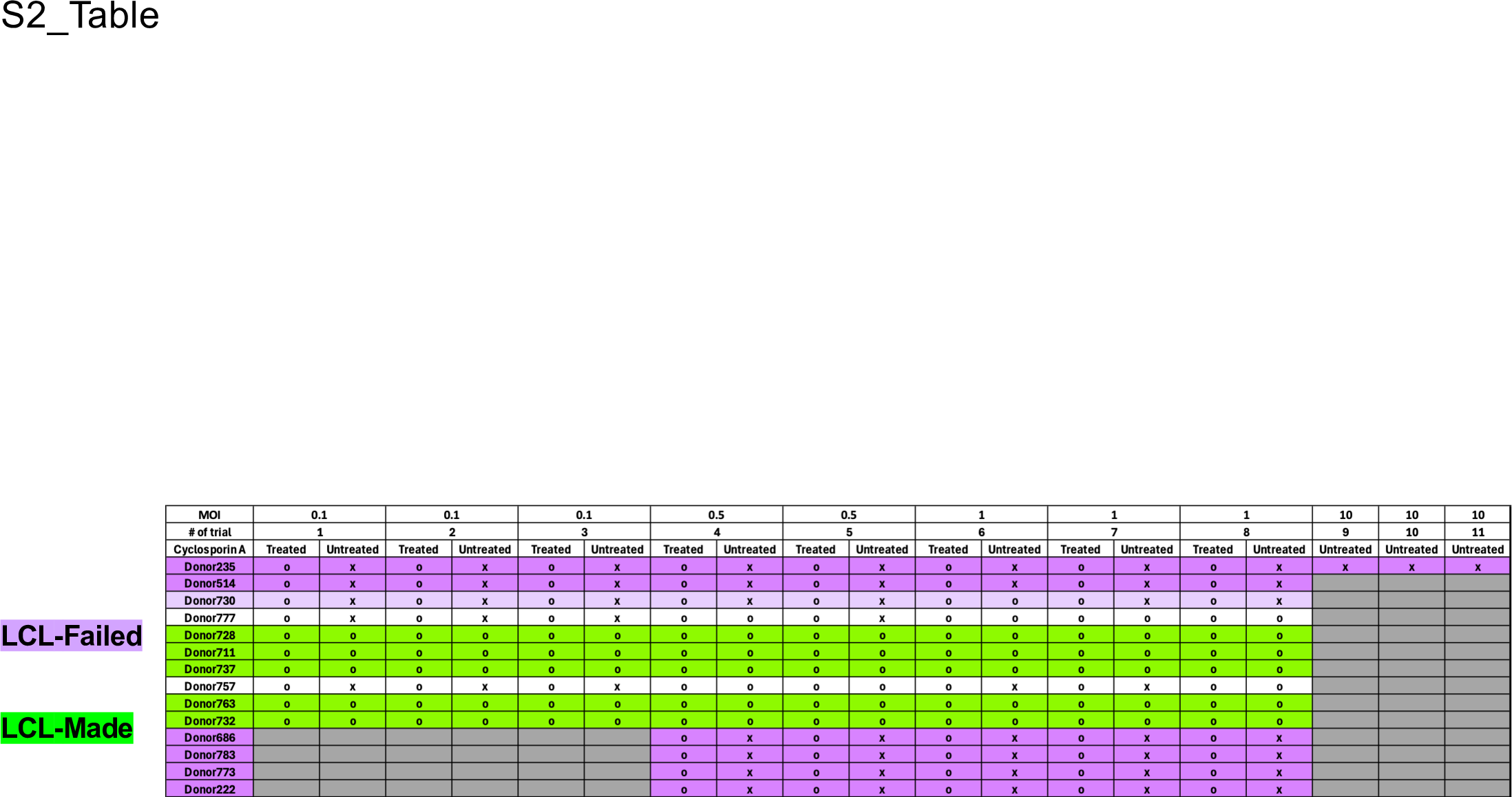

Supplement: S2 Table — Donor PBMCs were infected with EBV at varying multiplicities of infection (MOIs) ranging from 0.1 to 10, and LCL outgrowth was assessed across 5–11 independent trials. Each cell represents the outcome of a single trial under the indicated condition. Donors highlighted in green successfully formed LCLs (LCL Made), while those in purple failed to establish LCLs (LCL Failed) under cyclosporin A–untreated conditions. “o” indicates successful LCL outgrowth; “x” indicates failure. Gray-shaded columns represent MOI conditions not tested for the corresponding donor. (TIF) [file ppat.1013746.s002.tif]

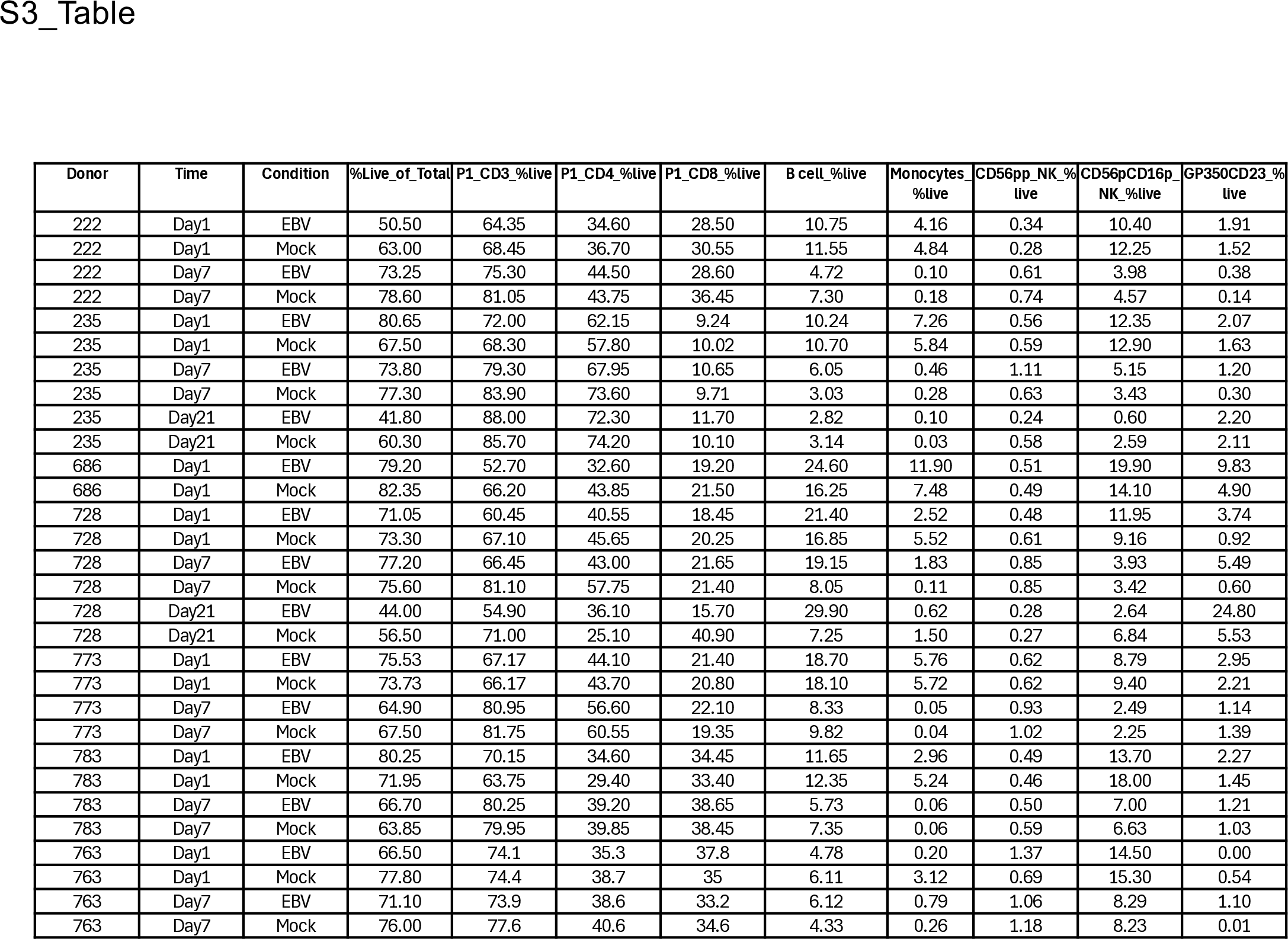

Supplement: S3 Table — Flow cytometry analysis of PBMCs from a subset of donors (n = 7). Day 1 and Day 7 timepoints represent the fully matched longitudinal dataset across these donors. Technical replicates were averaged per donor prior to analysis. For each donor, EBV-infected and Mock conditions are shown. Frequencies are expressed as percentage of live cells for CD3 ⁺ T cells (including CD4⁺ and CD8 ⁺ subsets), B cells (CD20⁺), Monocytes (CD14⁺ and CD64⁺), and NK cell subsets (CD56⁺⁺ and CD56 ⁺ CD16⁺). GP350 ⁺ CD23 ⁺ cells are shown as a percentage of live cells. Day 21 data are shown for the subset of donors where available. (TIF) [file ppat.1013746.s003.tif]

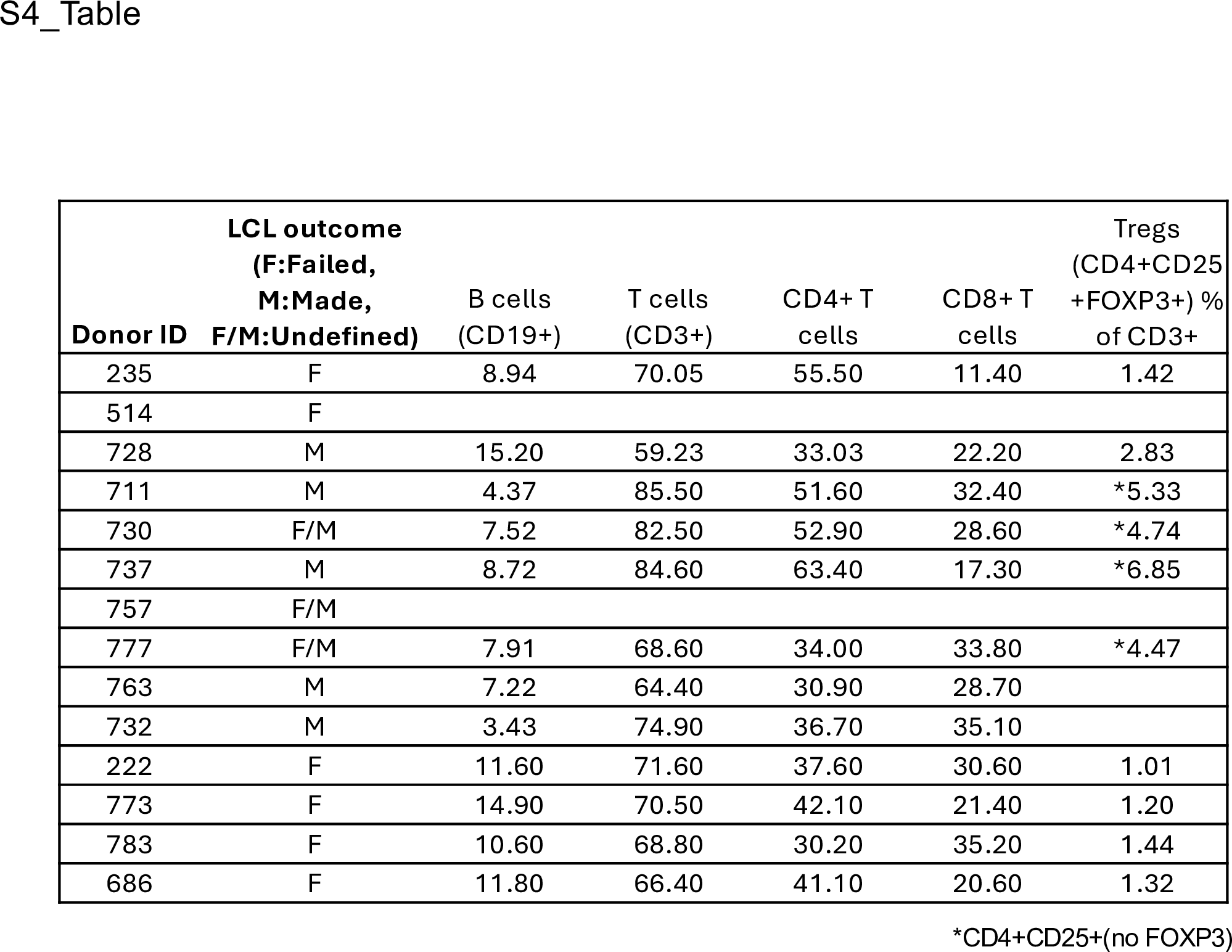

Supplement: S4 Table — Table summarizes baseline immune cell composition (% of live cells) measured prior to EBV infection. Columns show the LCL outcome, the frequencies of B cells (CD19⁺), total T cells (CD3⁺), CD4 ⁺ T cells, CD8 ⁺ T cells, and CD4 ⁺ CD25 ⁺ T cells (% of CD3 + T cells). Values with star* represent CD4 ⁺ CD25 ⁺ cells quantified without FOXP3 staining. (TIF) [file ppat.1013746.s004.tif]

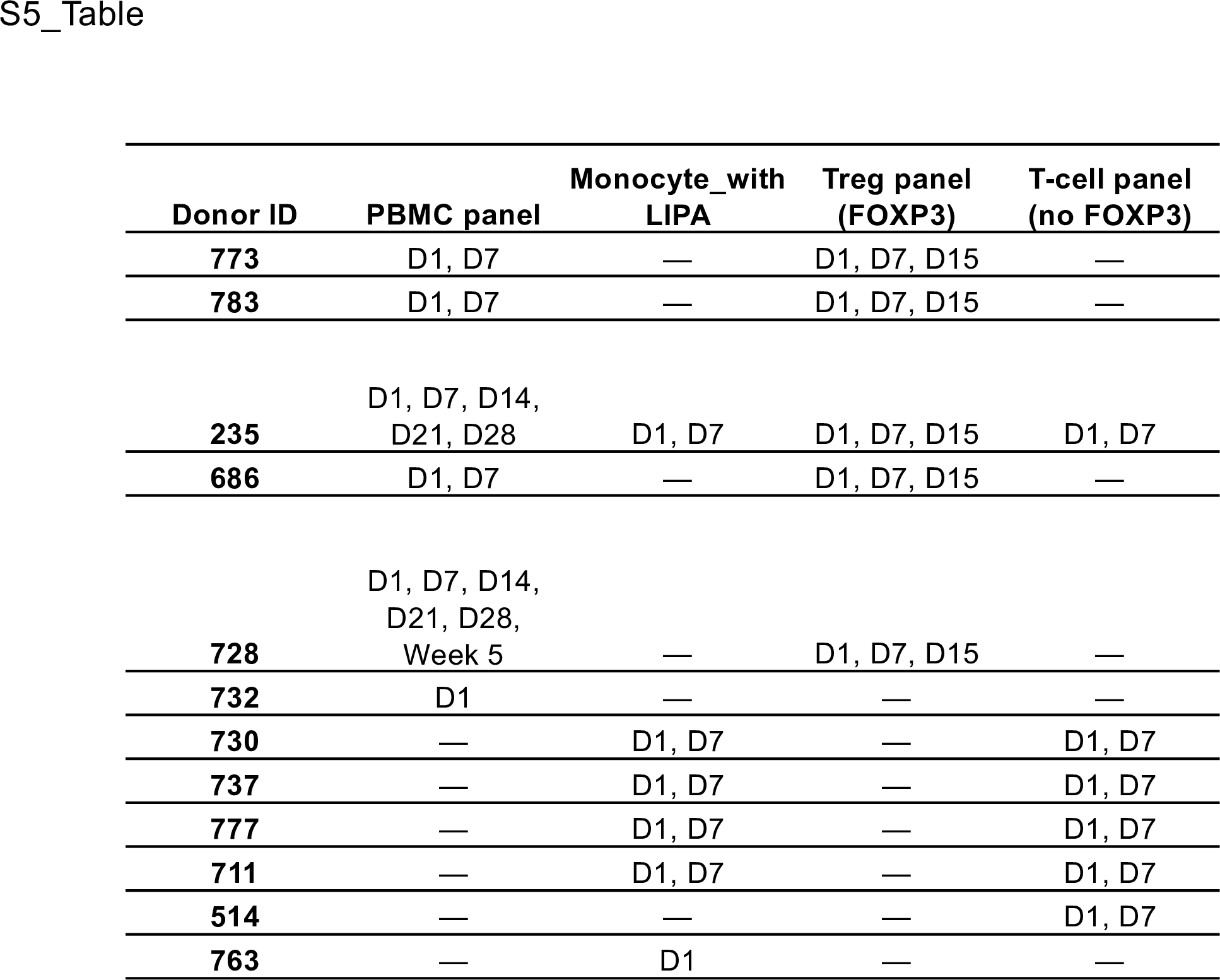

Supplement: S5 Table — This table summarizes inclusion of all donors across PBMC, Monocyte, Monocyte with LIPA, Treg (FOXP3⁺), and T-cell (no FOXP3) panels at each collection timepoint. PBMC analyses were conducted longitudinally up to Week 5, whereas Monocyte, Treg, and T-cell panels focused on early timepoints (Days 1–15). Dashes (—) indicate that a donor was not included in the specified panel. (TIF) [file ppat.1013746.s005.tif]

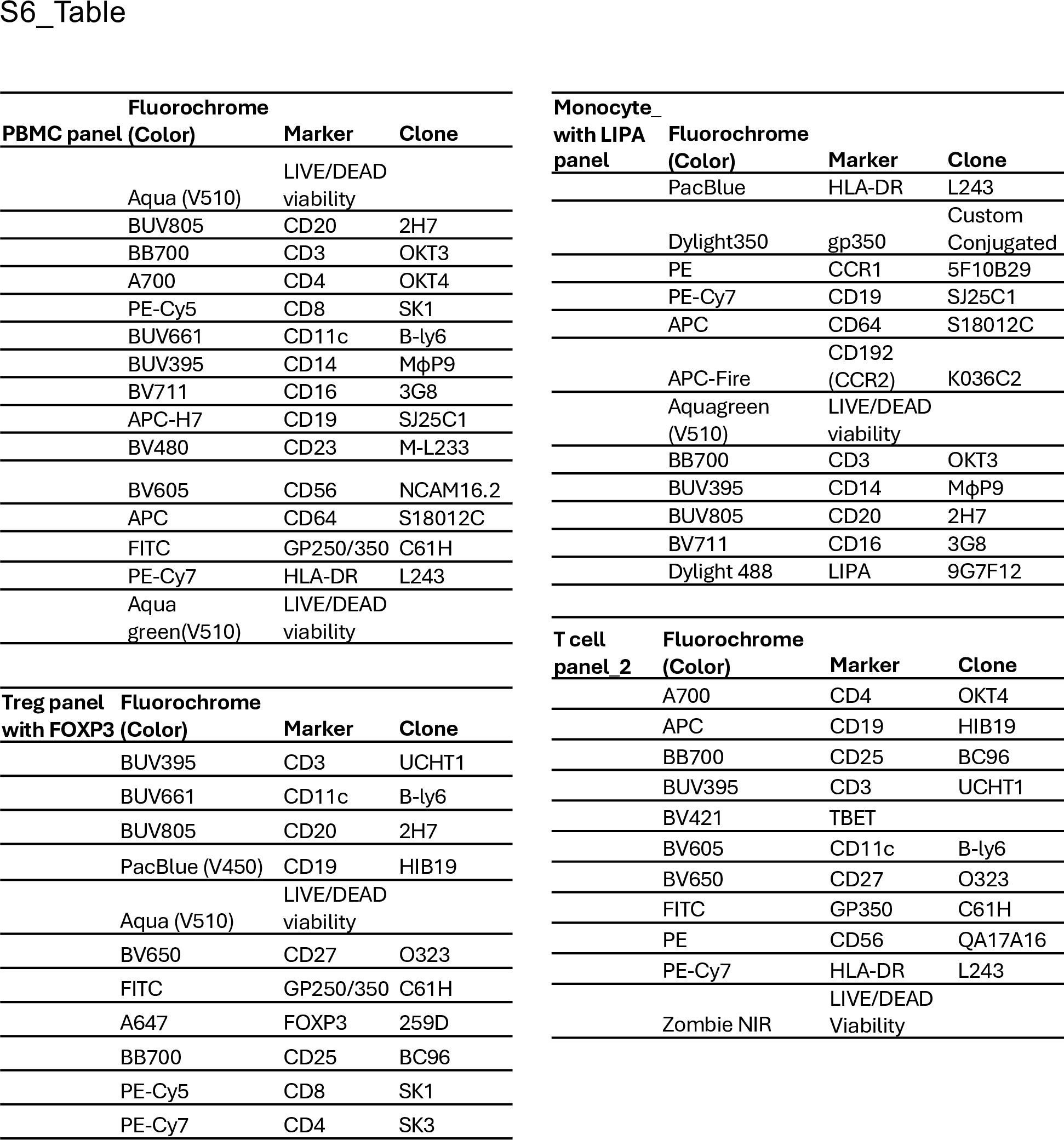

Supplement: S6 Table — This table lists all antibodies, fluorochromes, and viability dyes used in each experimental panel, including PBMC, Monocyte, Monocyte with LIPA, Treg with FOXP3, and T-cell no FOXP3 panels. Panels were optimized for multicolor analysis, and compensation controls were prepared for each fluorochrome using single-stained control samples. (TIF) [file ppat.1013746.s006.tif]

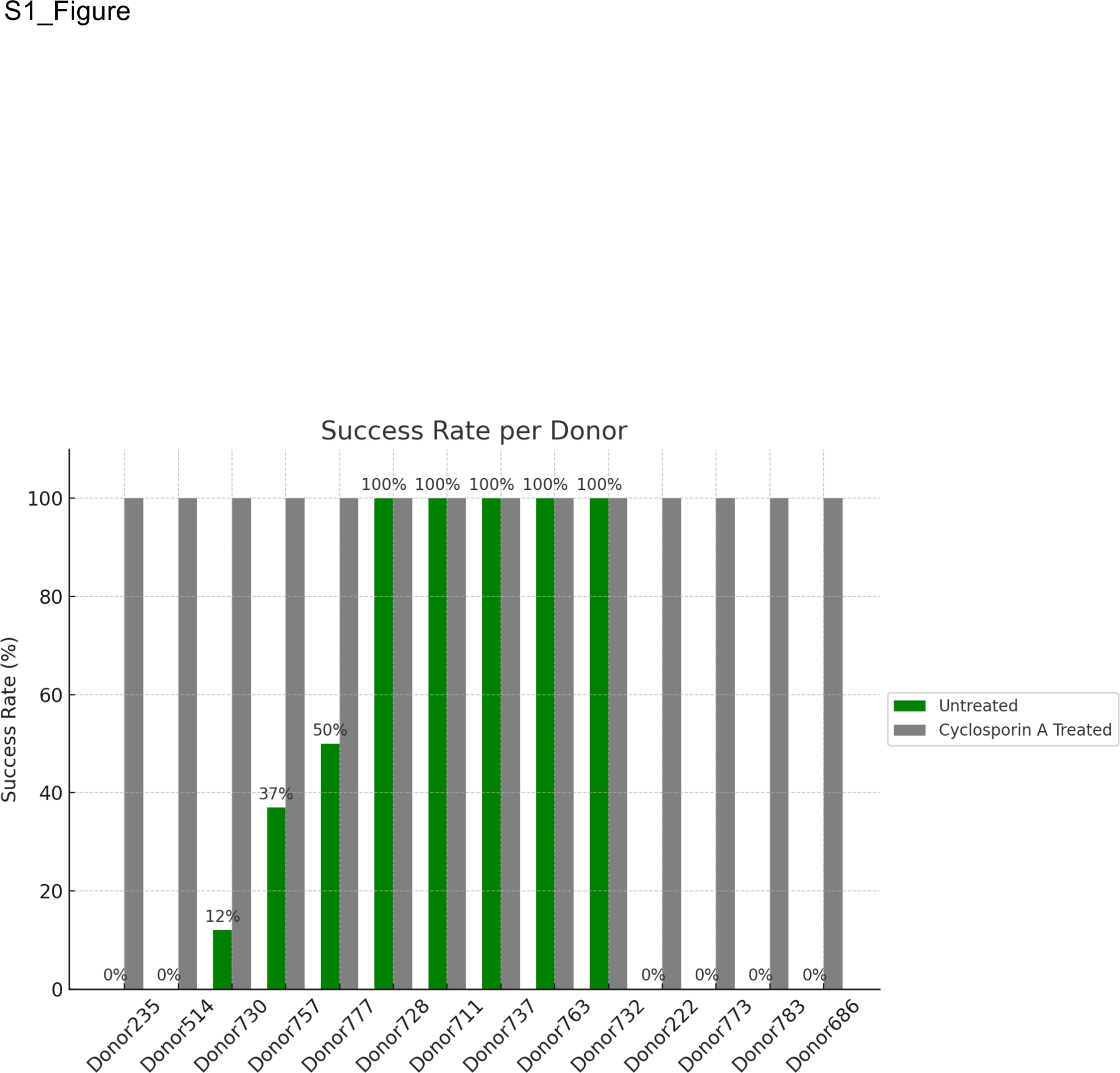

Supplement: S1 Fig — Bar plot shows the percentage of successful LCL outgrowths per donor under untreated (green) and Cyclosporin A–treated (gray) conditions. Each bar represents the proportion of successful EBV infections out of total attempts across multiple MOI conditions (see S2 Table). (TIF) [file ppat.1013746.s007.tif]

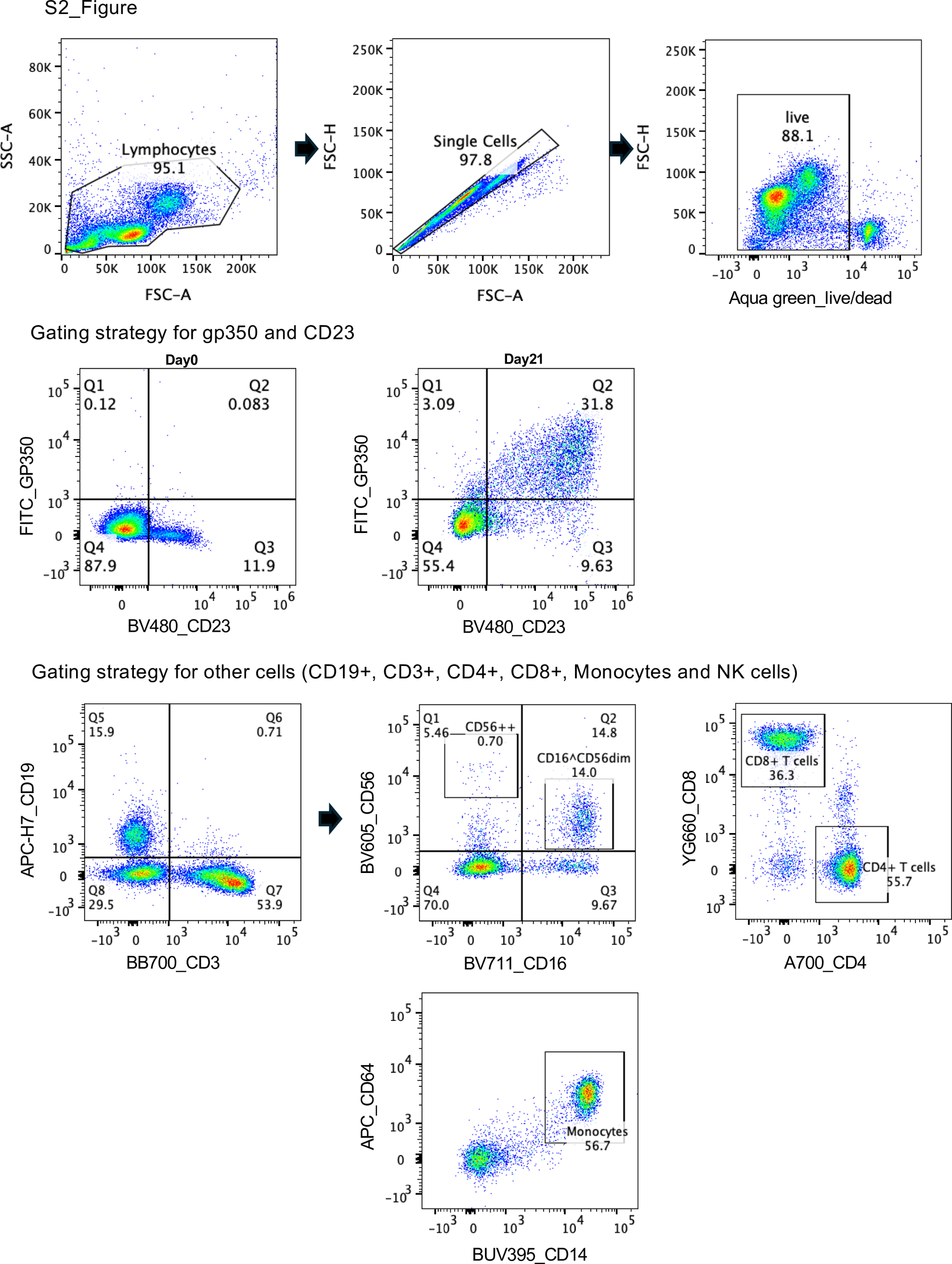

Supplement: S2 Fig — Cells were gated sequentially for lymphocyte population (FSC-A vs. SSC-A), single cells (FSC-A vs. FSC-H), and live cells (Aqua green Live/Dead dye). Lineage-specific markers were then applied within the live, singlet gate to define major immune subsets, including monocytes, T cells, NK cells, and B cells. (TIF) [file ppat.1013746.s008.tif]

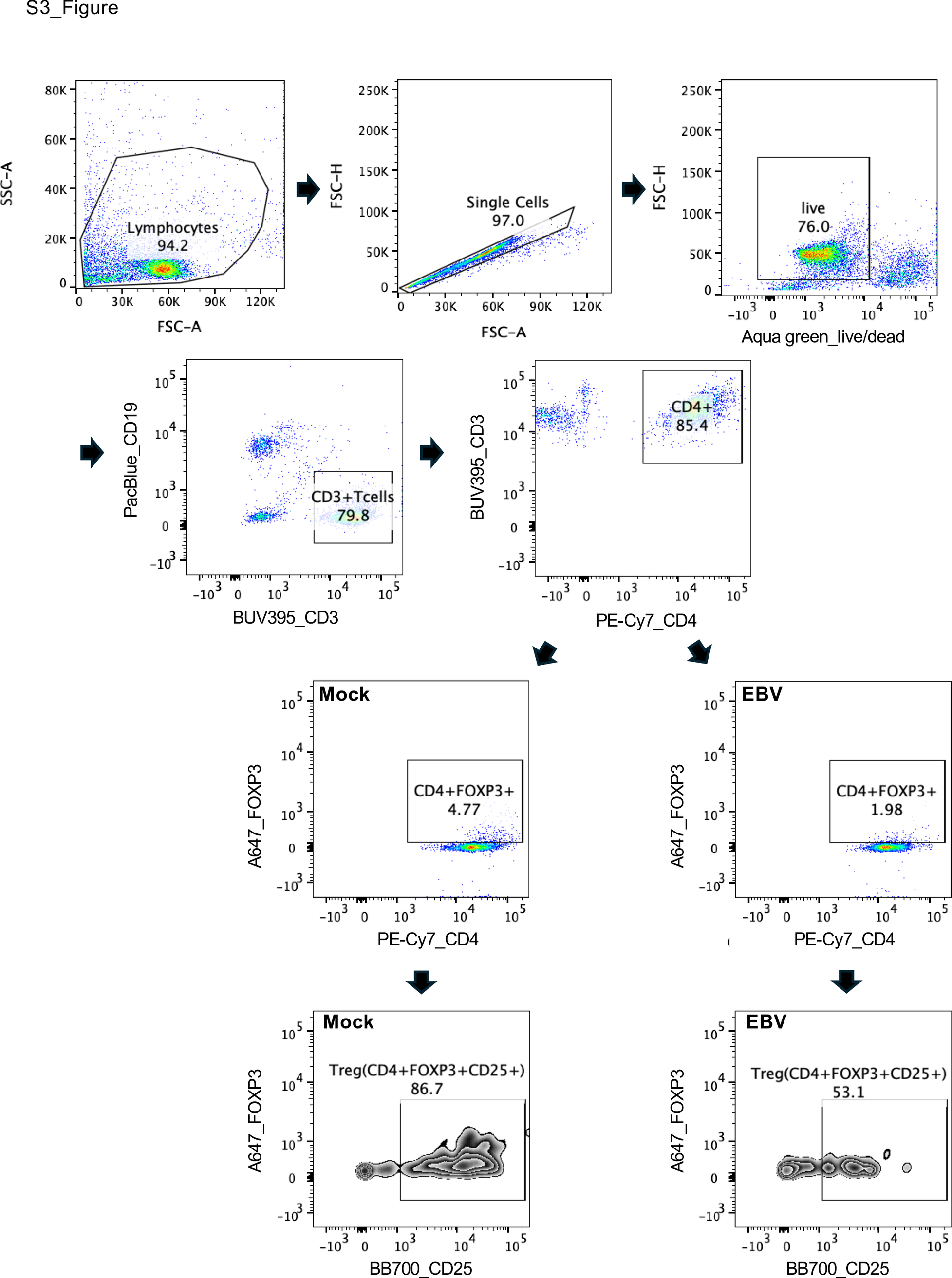

Supplement: S3 Fig — Flow cytometry plots showing sequential gating to identify CD4 ⁺ FOXP3 ⁺ CD25 ⁺ regulatory T cells. (TIF) [file ppat.1013746.s009.tif]

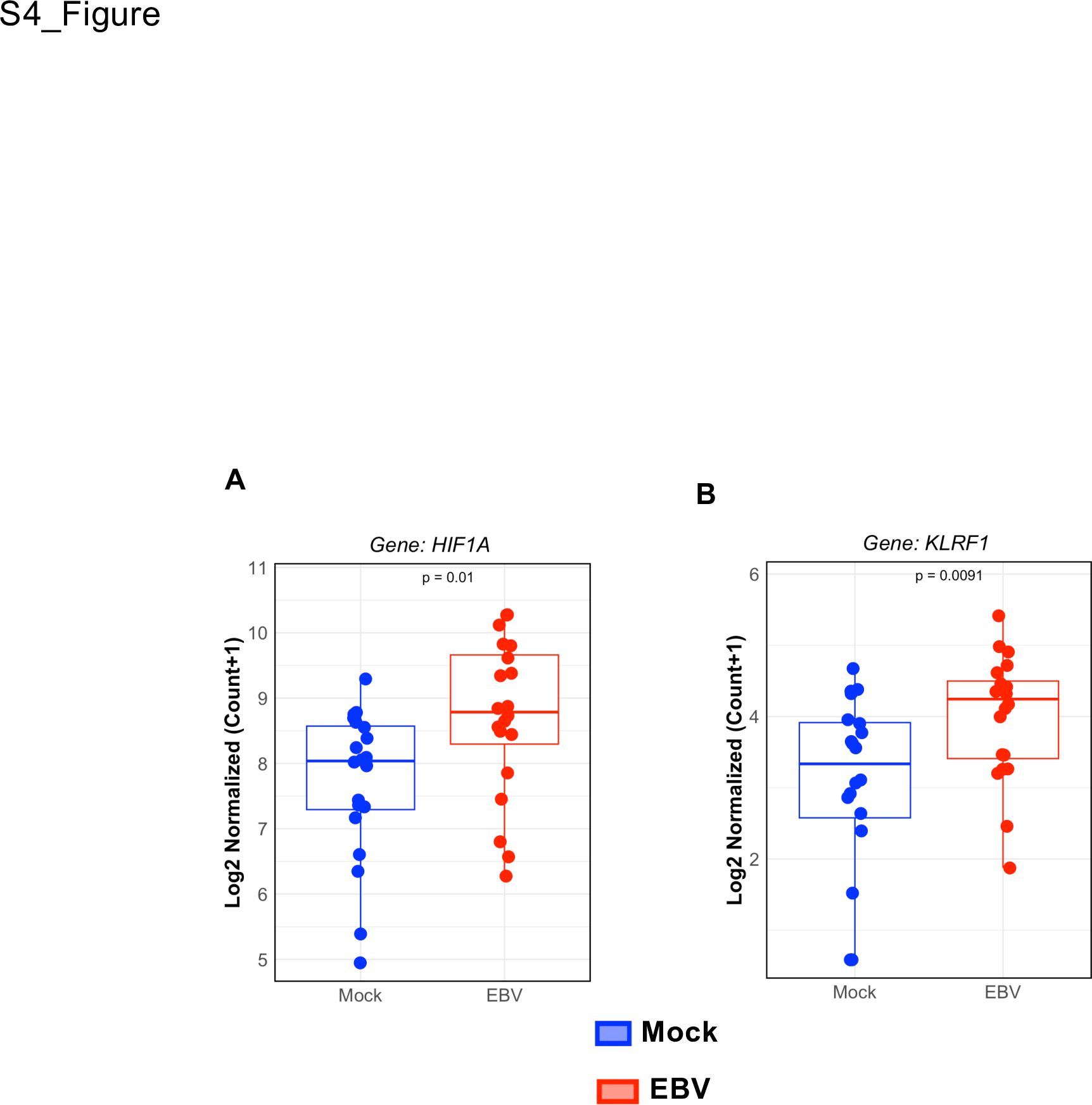

Supplement: S4 Fig — (A) Expression of HIF1A and (B) KLRF1 in PBMCs following ex vivo EBV infection, compared to mock-treated controls, across all timepoints (Days 1, 7, and 14). Boxplots show log2-transformed normalized gene counts. p-values were calculated using a paired Student’s t-test. (TIF) [file ppat.1013746.s010.tif]

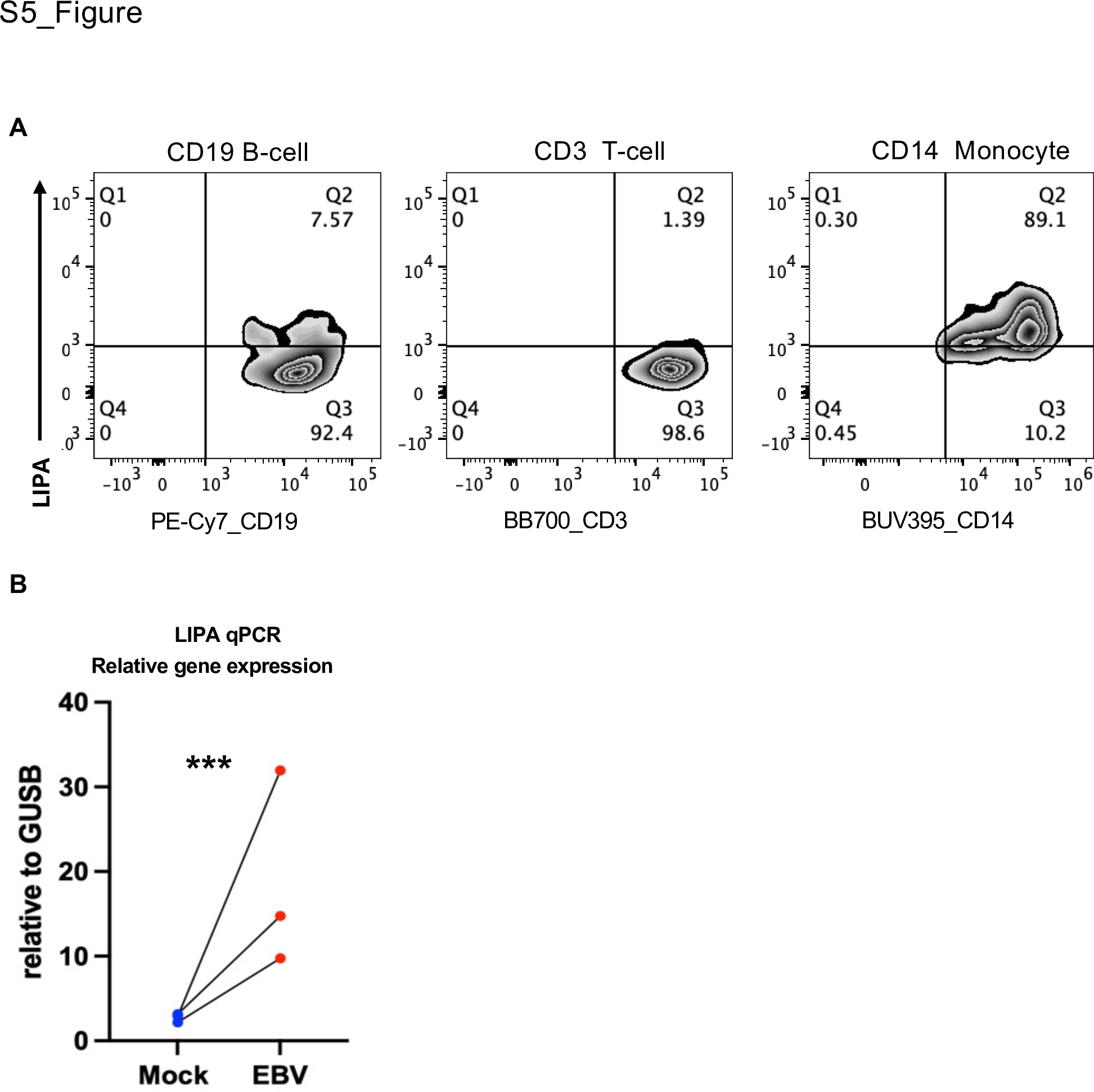

Supplement: S5 Fig — (A) Flow cytometry plots showing LIPA expression in CD19 ⁺ B cells (left), CD3 ⁺ T cells (middle), and CD14 ⁺ monocytes (right). (B) Relative LIPA mRNA expression measured by qPCR in PBMC infected with EBV compared to mock at Day1. Expression normalized to GUSB. ***p < 0.001 by a paired Student’s t-test. (TIF) [file ppat.1013746.s011.tif]

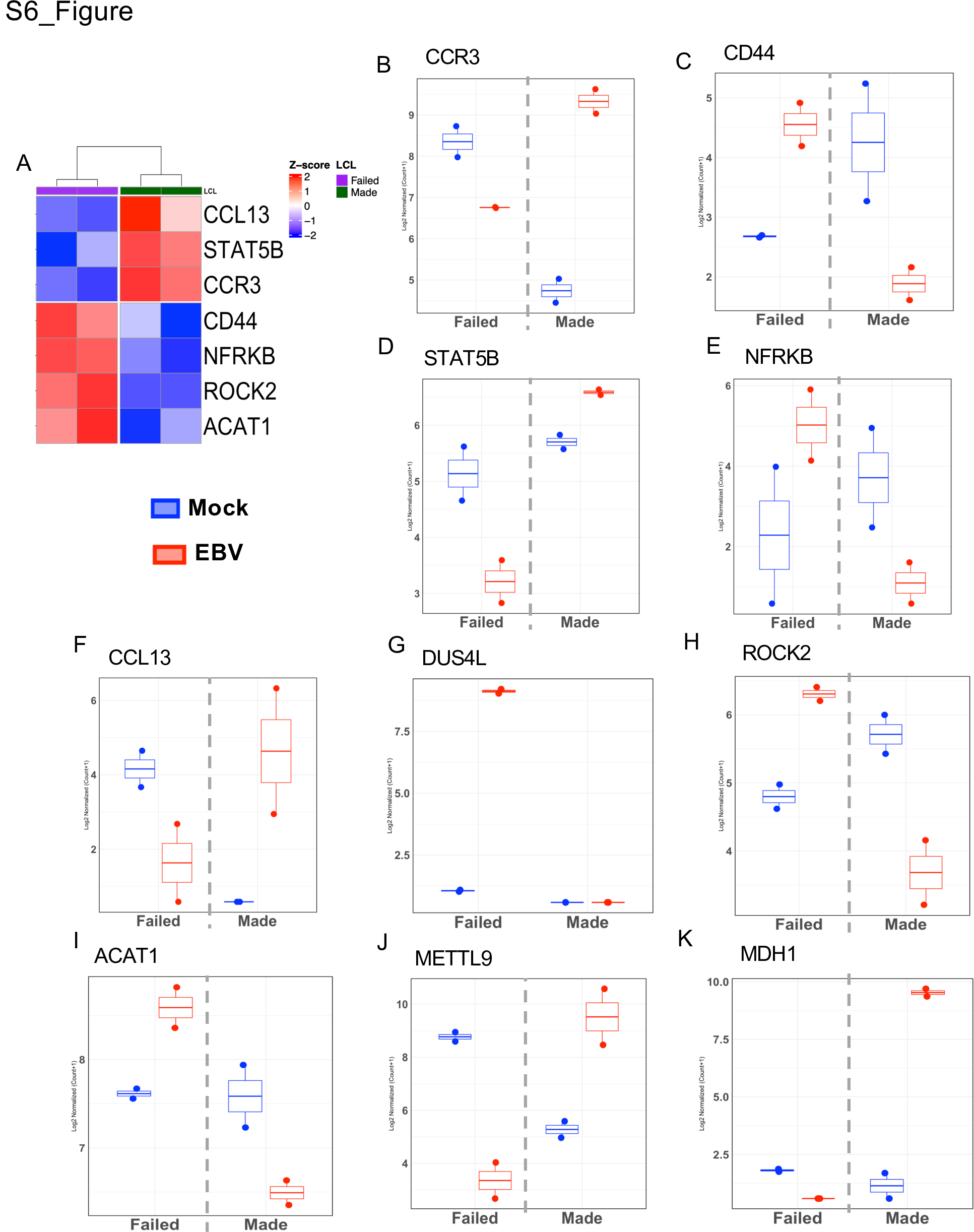

Supplement: S6 Fig — (A) Heatmap of selected differentially expressed genes (rows) related to immune activation and redox signaling, comparing LCL made (green) and failed (purple) donor groups at Day 14. Expression values are scaled by gene (Z-score). (B-K) Boxplots showing log-transformed normalized expression values of representative genes in EBV-infected and mock conditions for each group. (TIF) [file ppat.1013746.s012.tif]

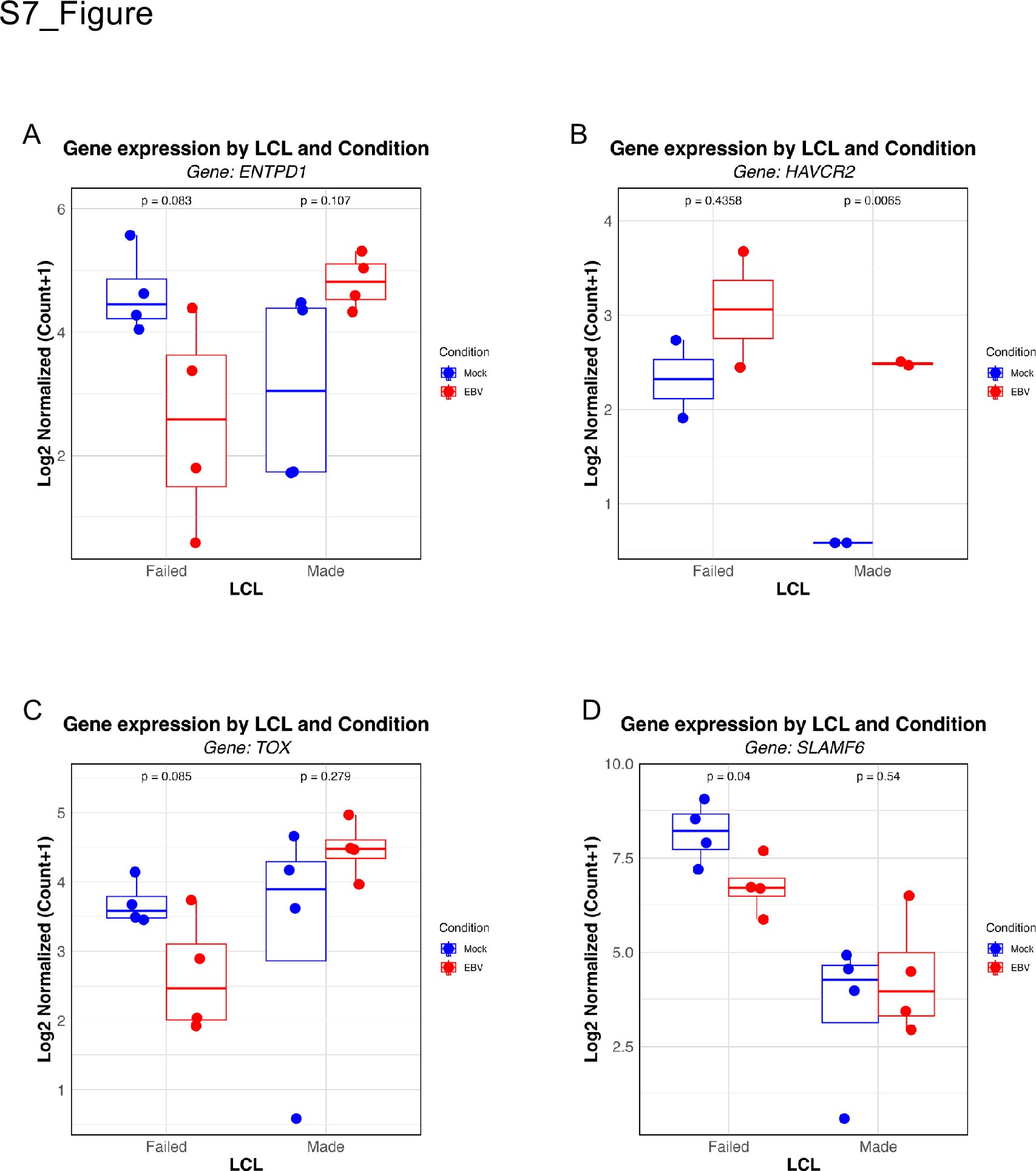

Supplement: S7 Fig — Boxplots display log₂ normalized expression values (counts +1) for each gene in peripheral blood mononuclear cells (PBMCs), stratified by donor LCL outcome (Failed vs Made) and infection condition (Mock [blue] vs EBV [red]). (A) ENTPD1, (B) HAVCR2, (C) TOX, (D) SLAMF6.p-values indicate results of unpaired two-tailed Student’s t-tests between EBV and mock conditions within each donor group. (TIF) [file ppat.1013746.s013.tif]

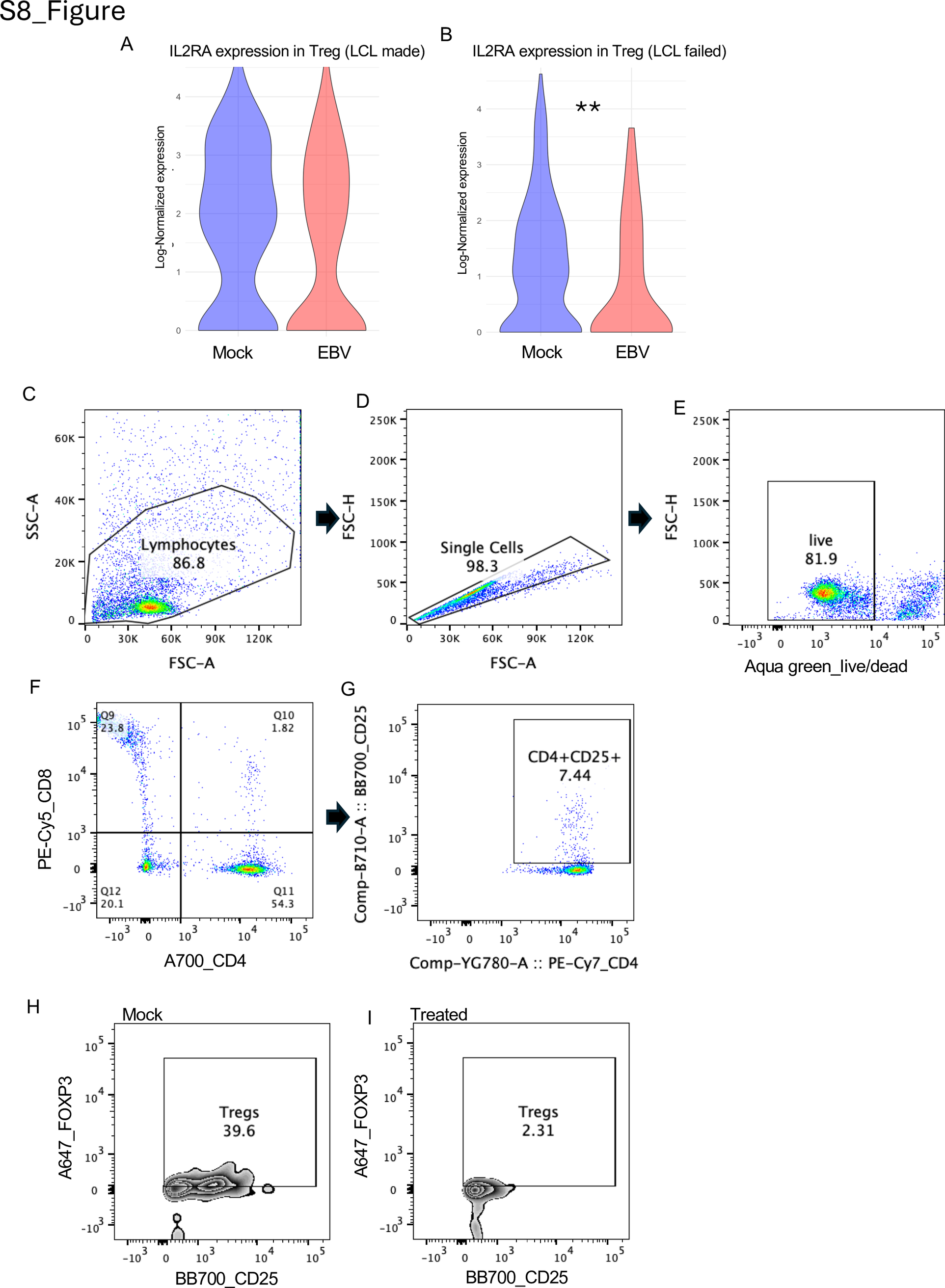

Supplement: S8 Fig — (A-B) Single-cell RNA-seq violin plots showing IL2RA expression in Treg clusters from LCL-made (A) and LCL-failed (B) donors under mock and EBV conditions. (C–E) Flow cytometry gating strategy: lymphocytes were first identified by forward and side scatter (C), followed by singlet discrimination (D), and viability gating using Aqua green live/dead stain (E). (F-G) CD4⁺ and CD8 ⁺ T cells were gated from live lymphocytes and CD25+ cells were gated. (H-I) Representative plots showing frequency of CD25 ⁺ FOXP3 ⁺ Tregs within the CD4 ⁺ CD25+ compartment in mock-IgG1 treated (H) and RG6292-treated (I) PBMCs. RG6292 selectively depleted CD4 ⁺ CD25 ⁺ FOXP3 ⁺ Tregs. (TIF) [file ppat.1013746.s014.tif]

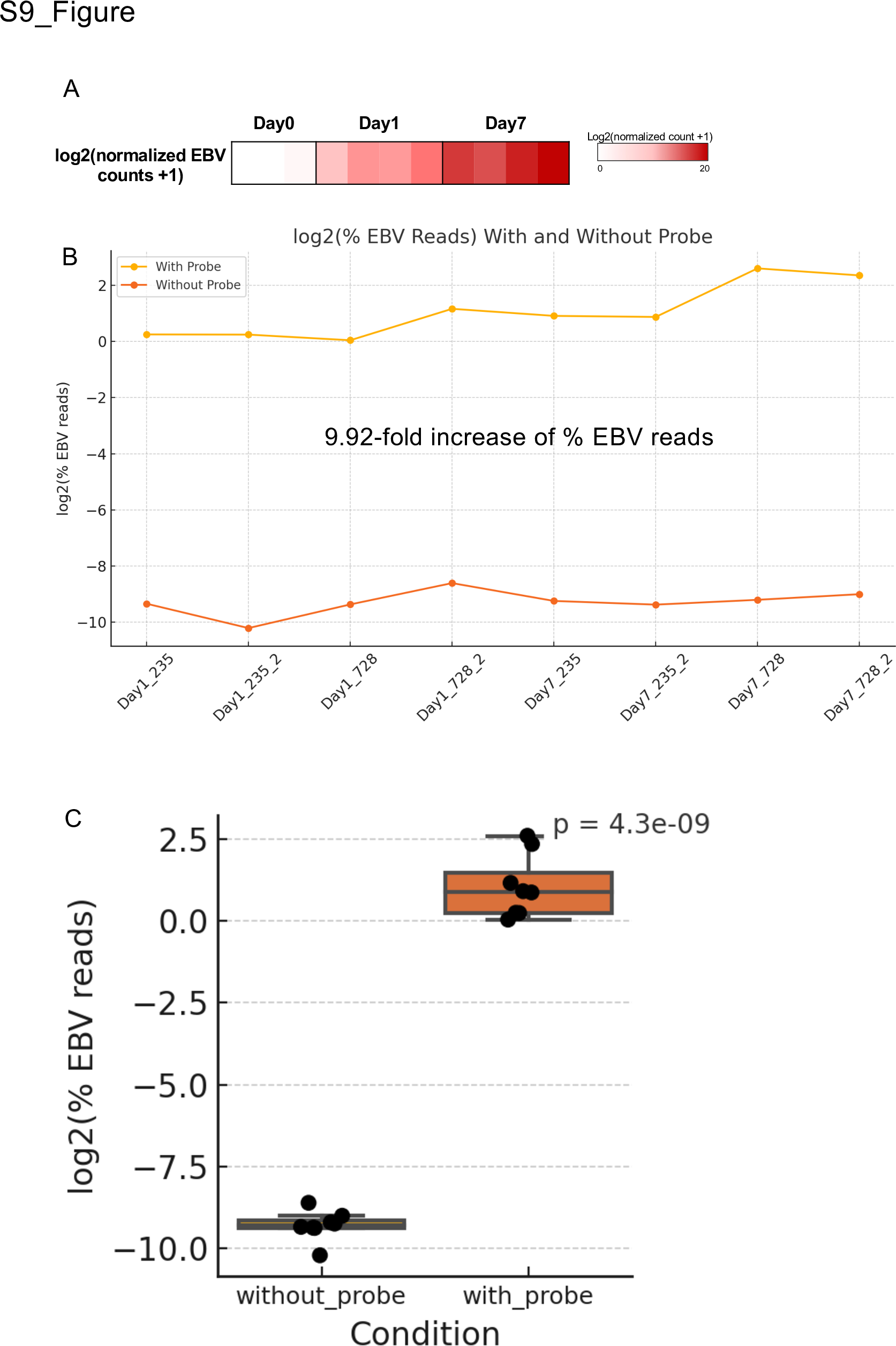

Supplement: S9 Fig — (A) Heatmap of normalized EBV counts (log₂ scale) across Day 0, Day 1, and Day 7 post-infection samples. (B) Line plot comparing log₂(% EBV reads) in samples with and without EBV probe enrichment across multiple donors and time points. (C) Boxplot of log₂(% EBV reads) showing a statistically significant increase in EBV reads in the with probe condition compared to without probe (paired Student’s t-test, p = 4.3e-09). (TIF) [file ppat.1013746.s015.tif]
